# Impact of Child and Family Factors on Caregivers’ Mental Health and Psychological Distress during the COVID-19 Pandemic in Greece

**DOI:** 10.3390/children11010007

**Published:** 2023-12-20

**Authors:** Dimitrios Papadopoulos

**Affiliations:** Department of Early Years Learning and Care, University of Ioannina, 45110 Ioannina, Greece; d.papadopoulos@uoi.gr

**Keywords:** developmental disability, children, caregivers, mental health, psychological distress, typical and atypical development, parental burden, family

## Abstract

Although primary caregivers of children with developmental disabilities (DDs) experience higher levels of distress than primary caregivers of typically developing children do, this problem has received limited attention in Greece. Therefore, this study examined mental health and associated factors among primary caregivers of children with and without DDs in Greece during the COVID-19 pandemic. This cross-sectional study included 156 participants. Primary caregivers completed a self-report survey on sociodemographic characteristics, the Depression, Anxiety, and Stress Scale-21 items, and the 12-item General Health Questionnaire. Primary caregivers of children with DDs, particularly mothers, reported more mental health problems and higher levels of psychological distress than the control group. Among families parenting a child with disabilities, caregivers’ psychological distress was significantly related to having a child with autism spectrum disorder and the severity of the child’s behavioral difficulties. Significant predictors of caregivers’ distress were the parent being female, the child being male, a single-parent family, a lower income, and higher depressive symptoms. Caregivers raising children with DDs face unique challenges in terms of care, necessitating the development of family-based interventions to improve the social-emotional well-being and overall quality of life for both parents and children.

## 1. Introduction

The ongoing COVID-19 pandemic has negatively affected the overall psychological health and well-being of both children and adults [1]. Children with developmental disabilities (DDs) experience impairments in multiple areas of development, including cognition, daily functioning, communication, emotions, and behavior [2]. Some of these children may need support and care from their parents and family throughout their lives, depending on their intellectual abilities, psychological status, or other factors. Individuals with disabilities are among the most vulnerable groups in terms of the pandemic’s effects [3,4] because their cognitive and intellectual limitations make it difficult for them to understand changes and restrictions, such as “stay-at-home” and “wear-a-mask” rules and guidelines that were provided to mitigate the spread of COVID-19 [5].

Raising a child with a DD is a multifaceted and challenging condition throughout the caregiver’s lifespan. This can lead caregivers to experience high levels of distress, poor quality of life, physical exhaustion, and a sense of continuous burden [6,7,8]. Furthermore, research on families with children with disabilities shows that caregivers not only feel more stressed than do the caregivers of typically developing children, but also experience chronic sadness, which is characterized by deep feelings of grief over the loss of the “child of their dreams” and their own expectations of their child’s future in general [9,10]. Even if these sorrowful feelings do not occur regularly, they are a normal result of raising a child who requires lifelong healthcare, individual treatments, and special education [11]. Similarly, qualitative research on parenting a child with a DD supports the notion that these caregivers experience feelings of sorrow, guilt, and being overwhelmed in reaction to the child’s diagnosis and the impact it has on their entire family life, including the marital quality of the parents and changes in family social life [8].

Previous studies conducted during the pandemic have revealed that, compared to the general population, families raising children with DDs have increased mental health problems, including symptoms of anxiety, post-traumatic stress, sadness, and poor sleep quality [12]. Children with DDs have long-term and specialized needs in terms of special education, individual treatments, and healthcare provision and were at risk of hospitalization during the pandemic outbreak [13]. Additionally, the intensity of child-externalizing behaviors amplifies the need for childcare and can reduce caregivers’ psychological well-being; caregivers must deal with the distress that arises from the ongoing demands associated with their role as primary caregivers, including continuous psychological adaptations, financial challenges, and heightened emotions [14]. In turn, because children spend a significant amount of time with their caregivers, the availability and wellbeing of the caregivers during the pandemic may also have had an indirect effect on children’s development and wellbeing. [15]. Evidence suggests that caregivers caring for a child with autism spectrum disorder (ASD) experience greater levels of distress than caregivers of children with other forms of DDs, such as attention deficit hyperactivity disorder (ADHD) and intellectual disability (ID), which can be driven by the severity of the child’s dysfunctional behavior [16].

During the COVID-19 pandemic, home quarantine and social isolation had a significant influence on all parents, as they faced numerous challenges in dealing with their children and managing their children’s needs solely at home. However, the impact on children with disabilities and their families was greater because of such children’s inability to comprehend and adjust to unexpected changes and new norms [15]. More importantly, the steps implemented to stop the spread of COVID-19 unexpectedly disrupted the delivery of therapeutic supports and educational or rehabilitation services and affected the daily routines of families that include children with disabilities, thereby heightening child-externalizing behaviors and parental stress levels [17]. Furthermore, such changes in children’s daily routines (e.g., changes in daily school education and transfers from regular to distance or online therapy and treatments) can result in the regression of developmental abilities and psychosocial skills that were obtained during treatment. This can lead to increased atypical behaviors and emotional challenges for both children and caregivers. Research suggests that changes in a child’s daily routine are linked to distress and a sense of instability not only in children but also in parents and other family members, which, in turn, are associated with elevated parental psychological distress [18].

Studies conducted during the pandemic have revealed a reduction in the mental health and quality of life of caregivers of children with DDs, indicating that caregivers faced additional psychological and social burdens [19]. This could be because parents devote more time and effort to caring for their children and responding to their children’s needs. In addition, the loss of family finances, social isolation, increased caregiving time, insufficient support services, a lack of clarity regarding the length of the lockdown, and uncertainty regarding their child’s future during the pandemic contributed to the stress experienced by parents of children with disabilities. Notably, increased parental stress can worsen related behavioral issues and negatively affect the psychological well-being of affected children, producing a vicious cycle [20]. Furthermore, the adverse effects of COVID-19 on the mental well-being of parents of children with DDs seem to be consistent across different cultures, with dysfunctional behaviors in children being linked to greater psychological distress in parents [21]. A notable determinant that adversely affected the psychological wellbeing of children with disabilities and their families during the pandemic was the restricted or limited accessibility of healthcare and social services for children with DDs [22]. This encompasses the interruption of mental health services and treatment sessions, such as speech, occupational, and physiotherapy, as well as the closure of educational institutions and outdoor activities. These disruptions could have long-term effects on children’s development and learning. Although many childcare facilities and clinics provide the option of online and distance telehealth services, families with limited income and education may encounter difficulties in accessing digital mental health treatments such as tele-rehabilitation, online therapy, and phone or distance consultation sessions [23]. Further, the increasing demand for pediatric hospitals to address COVID-19 cases has resulted in families with children who are at risk for complex developmental conditions losing or having to wait for months for their child’s assessment, screening, and availability of medical and supportive care services [24].

Research suggests that almost 80% of children with DDs commonly have psychiatric comorbidities and encounter mental health issues such as sleep disturbances, behavioral impairments, anxiety, and emotional disorders. In a recent study, Bouton et al. [24] examined the mental health status of children with neurodevelopmental disorders (NDD). They found increased scores on various scales of the Child Behavior Checklist in approximately 48% of preschool-aged children and 61% of school-aged children, suggesting that internalizing and externalizing behavioral difficulties were most common among children with NDD. Consequently, the persistence and escalation of these mental health issues result in stress and emotional fatigue, which have a detrimental impact on the health and well-being of both children and caregivers. While all children with DDs have an increased risk of acquiring mental health comorbidities compared to children with other DDs [25] and typically developing (TD) children [26], those diagnosed with ASD are especially prone to emotional and behavioral disorders [27]. These challenges frequently appear as unpredictable behaviors, such as tantrums, stereotyped and disruptive behaviors, and noncompliance [28]. Additionally, individuals with these mental health concerns typically have trouble with psychosocial adjustment and adaptive functioning. Moreover, the presence of mental health comorbidities may require medication to address the psychiatric symptoms in children. During the pandemic, caregivers were often required to increase their child’s medication dosage to address the child’s maladaptive behavior caused by the changes in routines and daily limitations [3].

Before the pandemic, studies examined the reciprocal relationships between stress and particular indicators of psychological well-being in households with children with disabilities. To the best of my knowledge, empirical research has generally neglected the complex relationship between individual and developmental components that predict the psychological wellness of caregivers raising children with DDs during the pandemic. To address this deficit, this study compared the mental health outcomes of caregivers of children with and without DDs and explored the factors associated with the psychological distress of caregivers of children with DDs during the second wave of the COVID-19 pandemic in Greece. I hypothesized that caregivers of children with DDs would experience more mental health difficulties and lower levels of well-being than parents of typically developing (TD) children, and that psychological distress would be predicted by caregivers and child-related factors. Some of these factors are the sociodemographic characteristics of both the caregivers and children, the type of disability and behavioral problems of the child, and the caregivers’ self-reported mental health. This study is valuable because it provides evidence of the unique needs of parents raising children with DDs. The results can be used by mental health professionals and government officials to create policies that reduce psychological distress and strengthen the resilience of parents and caregivers raising children with DDs.

## 2. Materials and Methods

### 2.1. Participants

A convenience sample of 156 caregivers from Greece was used (82 parents of children with DDs and 74 parents of TD children). There were 82 parents (*M_age_* = 36.75 years, *SD_age_* = 5.64; range = 27–50 years) with children with DDs ranging in age from 4 to 12 years (*M_age_* = 7.87 years, *SD_age_* = 2.67; 30% female). This age range was chosen to maintain sample homogeneity, as the concerns of parents with very young or adult children may differ greatly from those of parents with school-aged children. To be eligible to participate in this study, participants in the DD group were required to care for a child with a DD. ASD (38%) was the most commonly reported diagnosis in children with DDs, followed by ADHD (33%) and ID (24%). Most parents were mothers (76%) and predominately from Greece (87%). In addition, the parents were required to live in Greece and be comfortable completing online surveys. The parents of TD children were recruited as controls from normal nursery, primary, junior, and senior schools. Table 1 presents the characteristics of this study participants by group.

### 2.2. Study Design

This cross-sectional study was conducted by the Panhellenic Association of Mental Health for Children and Adults in Athens, Greece, a nonprofit organization that provides children and adolescents with individual treatment and support (e.g., speech and language therapy, occupational therapy, individual psychotherapy, and family therapy). All individuals who accepted special treatment were officially diagnosed by a multidisciplinary mental health team.

### 2.3. Procedures

Potential participants, namely all primary caregivers of children with DDs—having a diagnosis of ASD; ADHD; or ID—whose children attended the outpatient clinic of a mental health institution in Athens; were contacted by e-mail to participate in this study. During this study, the children with DDs received individual treatments, including speech therapy, occupational therapy, and psychotherapy support, either in person or online. The in-person option was introduced after the Greek government allowed special education institutions and outpatient clinics for children with any type of disability that remained open and continued to provide services to children and their families during the second phase of the COVID-19 pandemic. Although these parents did not experience an interruption in the support services provided to their children, only adjustments were made to the treatment schedule to reduce the duration of time that the children spent in the clinic’s waiting room. Therefore, this study did not address the possible effects of limited assistance from educational or other rehabilitative settings for children with DD. The electronic invitation to participate in this study included a link to an online survey with an informed consent form. To be eligible for inclusion, caregivers needed to fulfill the following criteria: (a) be the principal caretaker of at least one child aged 4–12 years; (b) be over the age of 18 years; (d) not have a history of mental illness; (e) be proficient in the Greek language; and (f) their child should have been diagnosed with ID, ASD, or ADHD. From a total of 104 primary caregivers who met this study criteria, 90 provided informed consent, and 82 completed all of the assessments (response rate = 91%). Caregivers were contacted in November 2020, and data were collected through January 2021. The caregivers of TD children were recruited as controls. To promote the survey, an online invitation with a link to the electronic questionnaire was posted on social media platforms such as Facebook and Twitter.

The families that consented to participate received two reminders to complete the survey. All parents voluntarily participated in this study and were not compensated for their time. This study was approved by the Ethics Committee of the Panhellenic Association of Mental Health for Children and Adults (approval number 654/05.10.2020) and performed in accordance with the principles outlined in the Declaration of Helsinki.

### 2.4. Measures

#### 2.4.1. Participants’ Sociodemographic Characteristics

The sociodemographic questionnaire asked for information on the parents’ gender, age, monthly household income, marital status, education level, employment status, and perceived social support. Information about the child’s gender, age, type of disability, and behavioral problems was also collected.

#### 2.4.2. Caregivers’ Mental Health Status

The Greek version of the Depression, Anxiety, and Stress Scale-21 items (DASS-21) [29,30] was used to evaluate caregivers’ mental health status. The DASS-21 is composed of 21 items designed to measure psychological distress and includes three subcategories: depression (e.g., depressed mood and loss of self), anxiety (e.g., fear of negative events), and stress (e.g., low frustration tolerance). Participants responded to each item using a four-point Likert scale, ranging from 0 (does not refer to me at all) to 3 (refers to me a lot/most of the time). The total depression, anxiety, and stress subscale scores are categorized as normal, mild, moderate, severe, and extremely severe. Higher scores indicate higher levels of mental health problems. The DASS-21 has been widely used among the Greek population and exhibits good reliability and validity [31]. In this study, the Cronbach’s alpha values for each subscale were as follows: depression, 0.83; anxiety, 0.79; and stress, 0.85.

The General Health Questionnaire (GHQ-12) is a self-report questionnaire designed by Goldberg et al. [32] to assess psychological health and wellbeing. The GHQ-12 includes 12 items with an equal number of positive and negative items asking the respondent to rate the degree to which they experienced a symptom in the previous week using a four-point scale: better than usual, same as usual, less than usual, or much less than usual; versus not at all, no more than usual, rather than usual, or much more than usual. This scale used a Likert scoring system with scores ranging from 0 to 3. The total score ranges from 0 to 36, with higher scores indicating poorer mental health. Scores lower than the cutoff of 12 are classified as clinical cases [33]. The GHQ-12 has been used as a screening tool in many studies across different cultures and has been translated into several languages. In addition, this instrument has good specificity, reliability, and reasonably high sensitivity [34,35]. In this study, the GHQ-12 had a satisfactory Cronbach’s alpha of 0.88.

### 2.5. Statistics

Categorical and scale variables were summarized using frequencies, percentages, mean values, and standard deviations (SDs). The significance of the differences between groups was evaluated using the chi-square test and the Student’s t-test for independent samples. Bivariate analyses were performed to gain initial insight into the interrelatedness between this study variables. Specifically, to assess the associations between continuous variables, Pearson’s correlation coefficient was used. One-way analysis of variance (ANOVA) and *t*-tests were performed to examine differences among this study variables.

Before the regression analysis was performed, categorical variables were converted to dummy variables as follows: gender was coded as 0 (male) or 1 (female); marital status was defined as married, divorced, or single or unmarried; monthly household income was divided into the three categories of low (less than 2000), medium (between 2001 and 3500), and high (more than 3500); employment status was categorized as working, not working, or working from home; caregivers’ education was categorized as secondary, post-secondary, or university degree; disability was divided into the three categories of ASD, ADHD, and ID; and the child’s behavioral problems were categorized as mild, moderate, or severe. The model includes age (in years) as one of the quantitative variables. Finally, for parents of children with DDs, linear regression analysis was performed to identify the predictors of psychological distress, as measured by the GHQ-12. The independent variables were caregivers’ characteristics (age, gender, education level, marital status, employment status, and income), children’s characteristics (age, gender, and severity of behavioral problems), type of disability (ASD, ADHD, and ID), and caregivers’ self-reported mental health status (depression, anxiety, and stress scores). The required sample size was calculated using G-Power for a fixed linear multiple regression model with an alpha value of 0.05 and a power of 0.80 [36,37]. The required sample size was 72; therefore, the sample of 82 participants included in this study is sufficient to examine the factors predicting psychological distress in caregivers of children with DDs. Data analyses were conducted using IBM SPSS 25.0, with a significance threshold of *p* < 0.05.

## 3. Results

### 3.1. Descriptive Statistics

The sociodemographic and clinical characteristics of the participants are presented in Table 1. Among the 156 caregivers who participated in this study, 82 (52.5%) had a child with a DD, and 74 (47.5%) had TD children. Regarding the type of disability, 51.2% of the children were diagnosed with ASD, 29.3% with ADHD, and 19.6% with mild ID. Among the caregivers, 75.6% were mothers, and more caregivers were in the 36–45 age range (35.9%) than in the other age ranges. No significant age- and gender-based differences were found between the groups (*p* > 0.05). More caregivers were married than divorced, although there was no statistically significant difference in the proportion between the two groups (*p* > 0.05). Most parents had completed a post-secondary degree (48.7%), and caregivers of TD children were slightly more likely to have achieved a higher level of education than parents of children with disabilities (40.5% vs. 31.7%). Compared to the control group, approximately 75% of caregivers of children with disabilities reported mild-to-severe difficulties with their children’s behavior (*p* < 0.001).

### 3.2. Caregivers’ Mental Health

The participants’ mean scores on the three dimensions (depression, anxiety, and stress) of the DASS-21 and GHQ-12, together with the mean scores of healthy controls, are presented in Table 2. Compared to the control group, caregivers of children with DDs scored significantly higher on the GHQ-12 (*t* = 8.51, *p* < 0.001), indicating a higher level of psychological distress. Considering each subscale of the DASS-21, caregivers of children with DDs scored significantly higher than the control group on depression (*t* = 6.35, *p* < 0.001), anxiety (*t* = 5.71, *p* < 0.001), and stress (*t* = 7.84, *p* < 0.001). With respect to gender (Table 3), mothers had higher levels of depression symptoms and stress (*p* < 0.001 in each case) in both types of families (those with a child with a DD and those with TD children). However, no significant gender-based differences were found in the scores for anxiety, indicating that parenting a child during the pandemic was stressful for both fathers and mothers. Regarding psychological distress, mothers raising children with DDs reported higher scores on the GHQ-12 than did fathers (*p* < 0.001).

### 3.3. Bivariate Analysis

The results showed that caregivers’ mental health scores for depression, anxiety, and stress (DASS-21) were significantly related to psychological health on the GHQ-12 (all *p* < 0.001), indicating that parents who had severe mental health problems had worse psychological health and poor well-being. In addition, caregivers’ DASS-21 and GHQ-12 scores were significantly associated with their household income, age, and education level (*p* < 0.001), as well as with the child’s age (*p* < 0.001). In terms of parents’ gender, females (compared to males) had higher scores for depression (*t* = −4.68, *p* < 0.001), stress (t = −3.73, *p* < 0.001), and psychological distress (t = −3.73, *p* < 0.001). Regarding the child’s characteristics, parents raising boys exhibited greater depression (*t* = 3.20, *p* = 0.002), stress (*t* = 2.73, *p* < 0.007), and psychological distress (*t* = 2.78, *p* < 0.006). Finally, among caregivers in the DD group, the child’s behavioral problems were significantly related to psychological distress (F(100, 931), *p* < 0.001), suggesting that caregivers of children with severe behavioral problems had elevated levels of psychological distress than parents of children with mild and/or moderate behavioral problems. Ranked by mean scores, severe behavioral problems (*M* = 17.96, *SD* = 3.09) ranked first, followed by moderate behavioral problems (*M* = 13.91, *SD* = 3.24) and mild behavioral problems (*M* = 10.79, *SD* = 1.64).

### 3.4. Regression Analysis

A linear regression model was used to predict psychological distress in families parenting a child with DD by considering caregivers’ and children’s individual characteristics as well as parents’ self-reported well-being features. Residual plots for the GHQ-12 and all independent variables, such as participant characteristics and scores on the depression, anxiety, and stress scales of the DASS-21, showed no violation of the linearity assumption. The results of the regression analyses are presented in Table 4. A strong relationship between predictors and global GHQ-12 scores was found, and the model explained 79.3% (adjusted R^2^) of the variance in caregivers’ psychological distress (F(13, 81) = 24.917, *p* < 0.001). The factors that significantly predicted the participants’ psychological distress, as measured by the GHQ-12, were caregivers’ gender, marital status, monthly income, and depression scores on the DASS-21, as well as the child’s gender and type of disability. Notably, the severity of children’s behavioral problems approached significance as a predictor of psychological distress (*p* = 0.07).

## 4. Discussion

This study, conducted during the second wave of the COVID-19 pandemic in Greece, is the first to compare the mental health status of caregivers of children with and without DDs and investigate the factors that impact psychological distress among caregivers of children with DDs. The overall results indicated that parent-specific factors of gender, marital status, income, and self-reported depression scores and child-specific factors of gender, type of disability, and severity of behavioral problems were significant predictors of caregivers’ psychological distress.

Examination of the DASS-21 and GHQ-12 questionnaires indicated that parenting a child with DD had a negative impact on the overall well-being of the caregivers’. DASS-21 and GHQ-12 scores were significantly higher among caregivers of children with DDs than in the control group, suggesting that these caregivers experienced an elevated level of psychological distress. Specifically, they encountered high levels of depression, anxiety, and stress and, in general, reported poor psychological health. These results are in line with previous research showing that caregivers of children with DDs are at risk of developing psychological and emotional problems [12,38,39,40], and these caregivers experience more parental stress and depressive symptoms than do parents of TD children [41,42]. Studies have suggested that during the COVID-19 pandemic, caring for children with DDs involved unique and complex experiences, such as dramatic changes in family routines and life plans. These changes typically raise concerns and influence future decisions, which may increase the risk of parents experiencing greater levels of psychological strain and further increase parenting demands [17,43].

Among the caregivers, those who raised children diagnosed with ASD had significantly higher psychological distress than parents raising children with ADHD or ID, indicating that the type of disability is a major contributor to psychological distress. This is in accordance with similar studies that reported increased levels of psychological distress and mental health problems in caregivers of children with ASD before and during the COVID-19 pandemic [44,45,46]. During the COVID-19 pandemic, Chen et al. [12] conducted a mental health survey using self-reported mental health measures with 1450 parents of children with disabilities. The findings indicated that caring for a child with intellectual or physical disabilities resulted in fewer mental health challenges than raising a child with ASD. Papadopoulos [8] interviewed nine mothers and found that they reported significant emotional, family, and social burdens related to caring for a child with autism. With respect to the associated behavioral problems and impaired communication skills seen in individuals with ASD, many factors have contributed to higher distress and poorer well-being in parents of children with ASD, including the often long diagnostic odyssey required to reach an official ASD diagnosis and caregivers’ experiences of autism-related stigma regarding public misunderstandings about the unique characteristics of individuals with autism [47]. Furthermore, the literature suggests that family members of individuals with ASD may exhibit symptoms of a broader autism phenotype, which includes modest and subtle ASD-like deficits in social communication, interaction with others, and repetitive interests and activities [48]. Consequently, some parents may experience difficulties that limit their ability to meet the complex needs of caring for their children.

This study revealed that depression is a significant indicator of caregivers’ psychological distress. Previous studies have revealed an association between distress and mental health problems in parents of children with DDs [49,50]. Depressive symptoms are associated with increased parenting distress, particularly in mothers. This may explain how withdrawal, lack of vital energy, feelings of victimization, guilt, contextual amplification, and a sense of disconnection contribute to experiencing higher psychological distress, which can affect a person’s thoughts, feelings, and behavior [14]. Recent studies have reported high levels of depression in parents of children with disabilities during the COVID-19 pandemic [12,51]. According to previous studies, regardless of the type of crisis (e.g., pandemic or economic crisis), a threat to one’s functioning, beliefs, and future goals, as well as the disparity between expectations, family resilience, external assistance, and subjective appraisal, has an equally strong impact on parents’ depression levels [44].

With respect to gender, this study found that, among the entire sample, mothers exhibited higher values of psychological distress than fathers, and similar trends were observed regarding mental health symptoms. Similar findings were found among families with children with DDs, indicating that mothers experienced greater psychological stress, anxiety, and depression than fathers, which is consistent with previous research [52]. Thus, while mothers and fathers of children with disabilities bear equivalent challenges, mothers may be more vulnerable to the stress and depression associated with the extensive caregiving responsibilities that are normally required when raising a child with a disability. Another possible explanation for this link is that sex hormones influence depression [53]. Mothers responsible for caring for children with disabilities may have health issues and develop mental health difficulties, such as depression. These findings imply that a unique aspect of the mother’s role in families with children with DDs contributes to greater psychological distress. Numerous theories have suggested that fathers’ and mothers’ parents differ in different ways [54]. For instance, psychoanalytical theory contends that mothers are children’s primary attachment figures and sources of emotional bonding, providing the child with psychological development [55], whereas fathers are typically less active in their children’s upbringing and are more distant from them. Gender and role theories ascribe disparities in child-rearing to distinct male and female traits, such as expressiveness and instrumentality. According to this notion, mothers are seen as nurturing icons, whereas fathers assume the roles of authoritative figures and providers for the family [56].

The results of this study suggest that the monthly income of parents of children with DDs influences the intensity of their psychological distress. Consistent with prior research on well-being and income [57,58,59], this study found that a family’s financial status is another significant predictor of parental distress. In families raising children with disabilities, a higher income was associated with greater psychological well-being and fewer mental health problems, indicating that a higher income may help caregivers continue to meet their children’s basic needs in terms of education, treatment, and healthcare [60,61]. Additionally, higher income could improve caregivers’ self-confidence in dealing with various life circumstances, such as the COVID-19 pandemic. In contrast, recent findings demonstrate that the pandemic caused some caregivers to lose employment or reduce working hours, resulting in difficulty in meeting the required expenses for their children, thereby increasing the financial burden and distress of caregivers [44]. Distress in this case may be explained by the fact that people with low incomes in relation to their educational attainment are often disappointed because of unfavorable social comparison effects, which could lower their well-being. Easterlin [62] asserted that income affects happiness and well-being not only because it enables a person to provide for their family needs but also because it acts as a social comparison function, allowing people to assess their own success or value in the community based on their wealth.

Marital status was a significant determinant of psychological wellbeing, with married caregivers faring better than divorced and unmarried caregivers, indicating that being married is a resilience factor in coping with caregiver stress, and spousal support is a particularly strong predictor of emotional well-being for these parents. Consistent with the results of this study, previous research has provided evidence that having a satisfactory and high-quality marital relationship serves as a significant source of support for overcoming difficulties in raising children with any type of DD [63]. It might be inferred that having a child with a disability while married may give both parents the chance to develop more loving, stable, and close relationships that foster better communication and a positive outlook for the family’s future. According to other studies of families of children with DDs, when caregivers’ marital satisfaction is low, they are more likely to experience psychological distress. However, several child and family characteristics, such as the type of disability, the child’s age, and the quality of the family relationship, can mediate this association. However, single-parent families face more challenges in raising children with disabilities because of social and financial disparities and difficulties in accessing services. Furthermore, the absence of another partner in the family with whom one can share the caregiving burden is associated with approximately three times the odds of experiencing distress when such a partner is present [53], which, in turn, may contribute to the implementation of negative parenting behaviors [64]. Moreover, research has consistently shown that single mothers experience approximately three times more depressive episodes than other groups [65].

Among child-specific factors, the gender of the child with DD was a powerful predictor of caregiver distress. This significant association is in line with the results of recent studies conducted in Europe and the Middle East. For example, Alhuzimi [66] investigated the stress levels and psychological well-being of 150 Saudi Arabian parents of children with disabilities during the COVID-19 pandemic. The results indicated that the child’s gender had a significant effect on parental stress and psychosocial wellness. Similarly, Bujnowska et al. [41] found that raising a son with a disability was significantly associated with caregiver distress and future anxiety among 167 caregivers of children with DDs. These findings can be attributed to two factors. First, communication deficits and maladaptive behaviors are more common in boys with disabilities than in girls with disabilities, which is linked to increased distress among caregivers. Second, from a social perspective, it is important to have children of a particular gender. Gender might lead to distinct assumptions about the social roles attributed to boys and girls, such as physical and/or intellectual superiority. This may increase parents’ fear of receiving negative assessments from others if their son is unable to fulfill such gender expectations.

Finally, the level of children’s behavioral problems was significantly associated with caregivers’ psychological distress. The caregiver’s GHQ-12 ratings increased as the severity of the child’s behavioral problems increased from moderate to severe. Children’s behavioral problems have consistently been found to be the strongest predictors of parenting stress [63]. Consequently, parents implement inappropriate parenting practices that affect childcare, which creates a vicious mental health cycle. This finding is supported by previous research, which suggests a bidirectional effect between care burden and caregivers’ skills for effectively coping with their child’s behavior; that is, caregivers’ negative emotional experiences weaken the parent-child bond, reduce parenting efficiency and well-being, and increase related behavioral issues [40]. Given that this study was conducted during the COVID-19 pandemic, this finding could be explained by the fact that nationwide home quarantine policies prevented children from accessing play and sport activities, as well as group-based interventions and treatments. This increased children’s screen time and loneliness in the absence of social activities and interactions with others [38,39]. These findings are consistent with recent literature showing that psychological distress in families caring for a child with a disability is more strongly associated with the level of behavioral problems than with the level of intelligence or functioning [18,67].

This study did not provide evidence of the effect of child age on caregiver distress, and these findings differ somewhat from those of previous research, which generally reported that older children with DDs are a significant factor in caregivers’ psychological health [68,69]. From a developmental perspective, as children with DD age, they may learn more effective emotional and behavioral regulation strategies, which in turn can positively influence caregivers’ psychological states, which may explain the aforementioned findings of prior research. In addition, most studies have reported inconsistent findings regarding the influence of caregivers’ age on caregiver care-burden-related stress. For example, a study conducted by Ha et al. [70] revealed that although psychological distress was reported among middle-aged and older primary caregivers of children with DD, the longitudinal impact of caring for a child with DD on psychological distress decreased as caregivers aged

### Strengths and Limitations

This study included responses not only from caregivers of children with a DD but also from a control group to compare the mental health status of families with and without a child with a DD.

Additionally, this study’s strength and novelty stem from the fact that it is the first study from Greece to examine the mental health of caregivers whose responses were obtained during the COVID-19 era, when many people’s lives were affected. Changes in the participants’ lives, such as household income and employment status at the time of this study, may have had an indirect impact on their self-reported mental health. However, the lack of data from the same sample during a time when there were no crisis was a limitation of this study. Thus, the results of this study are limited in terms of providing conclusions regarding the factors that predict psychological distress in families parenting children with DD under normal conditions. This study has several further limitations. First, this cross-sectional survey was limited with respect to establishing a causal relationship between the factors that predict distress in caregivers of children with DDs. Hence, it is crucial to conduct longitudinal research to evaluate, for instance, the causal relationships between child and family attributes and the symptoms of depression in caregivers of children with disabilities. Second, only three types of DDs were entered in the regression analysis after adjusting for child and family factors; thus, the results cannot be generalized to parents of children with other special needs. Third, a recall bias might have occurred because all outcomes were self-reported. Nonetheless, the use of self-report scales is frequently used in studies to assess mental health, owing to their convenience [12]. Because an online questionnaire was used in this study, the computer literacy of the respondents might have affected their responses. This study used a convenience sample of caregivers of children with DDs. Thus, the current findings should be interpreted carefully and cannot be easily generalized to other contexts and caregivers caring for a child with a different form of disability, such as a physical, sensory, or motor disability. Convenience sampling is widely accepted as a standard in the developmental and behavioral sciences because it is costly and impractical to use probability samples [17,71,72], especially during crises such as the COVID-19 pandemic. Furthermore, these findings pertain primarily to mothers, who are typically their children’s primary caregivers. Therefore, the results do not allow us to determine how different the impact was on fathers. Future studies are necessary to fill this gap and examine the impact of fathers’ roles in childcare because fathers’ emotional interactions and involvement have been repeatedly reported as a major influence on their children’s overall growth [73]. Finally, physical and mental health comorbidities and the intellectual status of children with DDs were not considered. Future studies should employ a longitudinal design with larger representative samples to examine other important variables that affect caregiver distress, such as the extent of caregivers’ social networks, support from relatives, and coping strategies. Further, future research should study the association between caregivers’ well-being and the clinical features and illness characteristics of children with DDs, as well as determine the impact of marital quality and family functioning on children’s development and well-being.

Moreover, future research should conduct in-depth interviews not only with caregivers but also with significant others, such as grandparents or healthy siblings associated with caring for a disabled child.

## 5. Conclusions

In summary, caregivers of children with disabilities reported elevated mental health problems during the COVID-19 pandemic, which may have negatively influenced the care provided. Overall, caregivers of children with ASD exhibited higher levels of psychological distress, anxiety, and depression than parents of children with other DDs and parents of TD children. There were remarkable differences in the mental health of all caregivers’ depending on their gender. Being a mother and being an unmarried/divorced parent with a lower income were significantly associated with greater psychological distress. Among child-specific characteristics, raising a boy with a DD and the severity of the child’s behavioral problems were common factors that predicted psychological distress. These findings could plausibly be due to COVID-19-induced abrupt routine changes causing decreased social and therapeutic supports and more frequent child behavioral problems. Thus, when face-to-face services are restricted for any reason (e.g., a pandemic crisis), the potential of employing technology (e.g., telehealth) to commence clinical activities for children with DDs and/or provide support to family members should be prioritized [17]. According to recent research, telerehabilitation, combined with in-person service delivery, is a novel and successful approach for treating the developmental and mental health problems of children with (DDs) when in-person care services are not available because of unpredictable conditions, such as the COVID-19 pandemic [74].

Regarding the clinical implications, the current study highlights the importance of families raising children with developmental disabilities to prioritize their psychological health. Public health policies should be adapted to address the unique needs of caregivers, who are more vulnerable and at a higher risk of developing mental health conditions associated with the continuity of care burden [8,11]. Furthermore, clinical studies of psychological interventions (e.g., family-based therapy with a focus on mindfulness and mentalization) and/or pharmacological treatments are needed for caregivers of children with developmental disabilities to reduce the incidence of psychological distress and depression symptoms and improve the overall quality of their lives.

## Figures and Tables

**Table 1:** Baseline socio-demographic characteristics of this study participants.

Variable	Total Participants (*n* = 156)	DD Family (*n* = 82)	TDC Family (*n* = 74)	*χ*^2^/*t*	*p*
Parents’ gender, *n* (%)					
Male	38 (24.4%)	18 (22%)	20 (27%)		
Female	118 (75.6%)	64 (78%)	54 (73%)	0.544	0.576
Parents’ age (years), *n* (%)					
26–35 years	52 (33.3%)	28 (34.1%)	24 (32.4%)		
36–45 years	56 (35.9%)	28 (34.1%)	28 (37.8%)		
46–55 years	48 (30.8%)	26 (31.7%)	22 (29.7%)	0.231	0.891
Parents’ marital status, *n* (%)					
Married	114 (73.1%)	64 (78%)	50 (67.6%)		
Divorced	36 (23.1%)	14 (17.1%)	22 (29.7%)		
Single/Unmarried	6 (3.8%)	4 (4.9%)	2 (2.7%)	3.763	0.152
Parents’ education level, *n* (%)					
Secondary education	24 (15.4%)	16 (19.5%)	8 (10.8%)		
Post-secondary education	76 (48.7%)	40 (48.8%)	36 (48.6%)		
University degree	56 (35.9%)	26 (31.7%)	30 (40.5%)	2.760	0.252
Family income per month, *n* (%)					
<2000 €	42 (26.9%)	38 (46.3%)	4 (5.4%)		
2001–3500 €	60 (38.5%)	26 (31.7%)	34 (45.9%)		
>3500 €	54 (34.6%)	18 (22%)	36 (48.6%)	34.270	<0.001
Employment status, *n* (%)					
Working	56 (35.9%)	28 (34.1%)	28 (37.8%)		
Not working	32 (20.5%)	18 (22%)	14 (18.9%)		
Working from home	68 (43.6%)	36 (43.9%)	32 (43.2%)	0.326	0.850
Child’s gender, *n* (%)					
Boy	98 (62.8%)	66 (80.5%)	32 (43.2%)		
Girl	58 (37.2%)	16 (19.5%)	42 (56.8%)	23.102	<0.001
Child’s age (years), *n* (%)					
4–6 years old	62 (39.7%)	34 (41.5%)	28 (37.8%)		
7–9 years old	52 (33.3%)	26 (31.7%)	26 (35.1%)		
10–12 years old	42 (26.9%)	22 (26.8%)	20 (27%)	0.266	0.875
Type of disability, *n* (%)					
ASD	42 (51.2%)	42 (51.2%)	0 (0%)		
ADHD	24 (29.3%)	24 (29.3%)	0 (0%)		
ID	16 (19.5%)	16 (19.5%)	0 (0%)		
Child’s behavioral problems, *n* (%)					
Mild	66 (42.3%)	18 (22%)	48 (64.9%)		
Moderate	44 (28.2%)	28 (34.1%)	16 (21.6%)		
Severe	46 (29.5%)	36 (43.9%)	10 (13.5%)	31.277	<0.001

**Table 2:** Caregivers’ mental health status according to group.

Variable	Total Participants (*n* = 156)	DD Family (*n* = 82)	TDC Family (*n* = 74)	*χ*^2^/*t*	*p*
Parents’ mental health status					
Depression, (M, SD)/*n* (%)	13.7 ± 3.9	15.4 ± 4.4	11.9 ± 2.1	6.353	<0.001
Normal	2 (1.3%)	0 (0%)	2 (2.7%)		
Mild	90 (57.7%)	34 (41.5%)	56 (75.7%)		
Moderate	56 (35.9%)	40 (48.8%)	16 (21.6%)		
Severe	2 (1.3%)	2 (2.4%)	0 (0%)		
Extremely severe	6 (3.8%)	6 (7.3%)	0 (0%)	25.320	<0.001
Anxiety, (M, SD)/*n* (%)	11.1 ± 2.8	12.2 ± 3.2	9.9 ± 1.6	5.713	<0.001
Mild	66 (42.3%)	24 (29.3%)	42 (56.8%)		
Moderate	74 (47.4%)	42 (51.2%)	32 (43.2%)		
Severe	14 (9%)	14 (17.1%)	0 (0%)		
Extremely severe	2 (1.3%)	2 (2.4%)	0 (0%)	21.908	<0.001
Stress, (M, SD)/*n* (%)	21.2 ± 5.1	23.8 ± 4.8	18.4 ± 3.8	7.841	<0.001
Normal	8 (5.1%)	0 (0%)	8 (10.8%)		
Mild	46 (29.5%)	12 (14.6%)	34 (45.9%)		
Moderate	62 (39.7%)	34 (41.5%)	28 (37.8%)		
Severe	40 (25.6%)	36 (43.9%)	4 (5.4%)	44.409	<0.001
GHQ-12, (M, SD)/*n* (%)	13.8 ± 3.9	15.9 ± 3.9	11.5 ± 2.4	8.518	<0.001
Non-cases	58 (37.2%)	16 (19.5%)	42 (56.8%)		
Cases	98 (62.8%)	66 (80.5%)	32 (43.2%)	23.102	<0.001

**Table 3:** Gender differences and caregivers’ mental health status.

	DD Family				TDC Family			
	Male	Female	*t*	*p*	Male	Female	*t*	*p*
Depression	10.4 (0.94)	12.41 (2.22)	−5.455	<0.001	12.78 (4.02)	16.09 (4.32)	−2.921	0.005
Anxiety	9.9 (1.8)	9.89 (1.51)	0.025	0.981	10.89 (3.05)	12.53 (3.18)	−1.954	0.054
Stress	15.9 (2.77)	19.3 (3.72)	−3.709	<0.001	21.67 (4.87)	24.38 (4.64)	−2.165	0.033
GHQ-12	9.5 (1.32)	12.19 (2.3)	−6.248	<0.001	14.67 (3.91)	16.22 (3.94)	−1.478	0.143

**Table 4:** Factors predicting psychological distress in caregivers of children with disabilities as measured by the GHQ-12 (*n* = 82).

	B	SE B	*β*	*t*	*p*	95% CI	F (13,81)	R^2^	Adj R^2^
Parents’ gender	−1.120	0.580	−0.118	−1.933	0.057	−2.277 to 0.036	24.917	0.826	0.793
Parents’ age	−0.757	0.677	−0.156	−1.117	0.268	−2.108 to 0.595			
Marital status	−1.509	0.595	−0.208	−2.536	0.014 *	−2.697 to −0.322			
Education level	−0.868	0.479	−0.155	−1.812	0.074	−1.824 to 0.088			
Monthly income	−3.005	0.496	−0.579	−6.063	<0.001 **	−3.994 to −2.016			
Employment status	−0.074	0.330	−0.017	−0.225	0.823	−0.733 to 0.585			
Child’s gender	1.721	0.663	0.173	2.595	0.012 *	0.398 to 3.044			
Child’s age	0.511	0.588	0.106	0.869	0.388	−0.663 to 1.685			
Type of disability	0.714	0.337	0.141	2.118	0.038 *	0.041 to 1.387			
Child’s behavioral problems	0.961	0.532	0.191	1.805	0.075	−0.101 to 2.023			
Depression	0.200	0.085	0.225	2.363	0.021 *	0.031 to 0.369			
Anxiety	0.039	0.095	0.031	0.407	0.685	−0.151 to 0.228			
Stress	−0.017	0.074	−0.021	−0.236	0.814	−0.165 to 0.13			

* *p* < 0.05, ** *p* < 0.001.

## Data Availability

The data presented in this study are available on request from the corresponding author. The data are not publicly available due to privacy and ethical restrictions.
